# The Health, Enlightenment, Awareness, and Living (HEAL) Intervention: Outcome of an HIV and Hepatitis B and C Risk Reduction Intervention

**DOI:** 10.3390/ijerph13100948

**Published:** 2016-09-24

**Authors:** Tabia Henry-Akintobi, Nastassia Laster, Jennie Trotter, DeBran Jacobs, Tarita Johnson, Tandeca King Gordon, Assia Miller

**Affiliations:** 1 Department of Community Health and Preventive Medicine, Morehouse School of Medicine Prevention Research Center, 720 Westview Drive SW, Atlanta, GA 30310, USA; tgordon@msm.edu; 2 Office of Public Health Preparedness and Response, Centers for Disease Control and Prevention, 1600 Clifton Road Atlanta, GA 30329, USA; nlaster@cdc.gov; 3 Wholistic Stress Control Institute, Incorporated, 2545 Benjamin E Mays Drive, Atlanta, GA 30311, USA; jt@wholistic1.com (J.T.); tjohnson@wholistic1.com (T.J.); 4 University of Alabama at Birmingham School of Education, 1720 2nd Avenue South Birmingham, AL 35294, USA; djacobs@uab.edu; 5 McKing Consulting Corporation, 2900 Chamblee Tucker Road, Building 10, Suite 100, Atlanta, GA 30341, USA; amiller@msm.edu

**Keywords:** health disparities, HIV/AIDS risk reduction, hepatitis risk reduction

## Abstract

African American women have among the highest HIV/AIDS and hepatitis B and C incidence rates in the United States, especially among those homeless or incarcerated. The objective of this study was to evaluate the Health Enlightenment, Awareness and Living Intervention, designed to decrease HIV/AIDS, hepatitis and related risky behaviors. The thirteen-session intervention was implemented among homeless and formerly incarcerated low-income African American women, ages 18 to 55, in Atlanta, Georgia from 2006 to 2010. A single group repeated measures study design was employed and consisted of a pre-test (n = 355) group, an immediate post-test (n = 228) group with a response rate of 64%, and a six-month follow up (n = 110) group with response rate of 48%, completing a 135-item survey. Paired-sample t-tests, McNemar tests, and repeated measures ANOVA were applied to compare survey results. Participants demonstrated statistically significant increases in hepatitis B and C knowledge over time (*p* < 0.001). Statistically significant decreases were also reported for unprotected sex in exchange for money, drugs or shelter (*p* = 0.008), and sex under the influence of drugs or alcohol (*p* < 0.001). Reported substance use decreased with statistical significance for alcohol (*p* = 0.011), marijuana (*p* = 0.011), illegal drugs (*p* = 0.002), and crack/cocaine (*p* = 0.003). Findings broaden the evidence base related to the effectiveness of HIV/AIDS and hepatitis risk reduction interventions designed for homeless and previously incarcerated African American women.

## 1. Introduction

African American women are at an increased risk for contracting sexually transmitted infections (STIs), including HIV/AIDS and hepatitis. At the end of 2013, of the total estimated number of women living with diagnosed HIV/AIDS, 61% were African American, 17% were white, and 17% were Hispanics/Latinas [1]. In addition, the rate of new HIV infections among African American women is 20 times higher than white women and almost five times higher than that of Hispanic/Latina women [2]. African Americans also have the highest incidence rate of hepatitis B compared to other ethnic groups, and African American women are 1.6 times more likely to die from viral hepatitis as compared to non-Hispanic whites [1,3]. According to recent research, approximately one-third of people infected with HIV/AIDS are co-infected with hepatitis B (HBV) or hepatitis C (HCV) [1]. Though HIV, HBV, and HCV can all be transmitted by direct blood-to blood contact, all three viruses have slightly different transmission patterns. In the USA, HIV is spread mainly by having anal or vaginal sex with someone who has HIV without using a condom or taking medicines to prevent or treat HIV, by the sharing of needles and drug injecting materials [1,2,3]. HBV is transmitted through activities that involve percutaneous or mucosal contact with infectious blood or body fluids (e.g., semen, saliva), including sex with an infected partner, injection drug use that involves sharing needles, syringes, or drug-preparation equipment, sharing items such as razors or toothbrushes with an infected person [1,2,3]. Transmission routes of HCV are injection drug use with needle sharing, occupational exposure in health care workers, contaminated blood products, transplants, and use of medical and paramedical devices [4,5,6]. HCV transmission by unprotected sexual contacts remains controversial [4].Prevalence rates of HIV/AIDS, HBV and HCV infection are highest among incarcerated African American women [7,8]. Substance abuse, including injection drug use (IDU), having sexual partners who practice IDU, sex in exchange for money, food or shelter are often precursors of incarceration for women, with higher rates among African American women [9,10,11,12,13,14,15,16]. In 2013, 113 out every 100,000 African American women were incarcerated compared to 51 out of every 100,000 white women [17]. These statistics express the urgency of established, effective interventions that are tailored to these populations.

Higher rates of HIV/AIDS among African American women in the United States have catalyzed national support and implementation of risk reduction interventions to address HIV/AIDS and co-occurring behaviors including substance abuse. Federally agencies including the Centers for Disease Control and Prevention (CDC), the National Institutes of Health (NIH), and the Health Resources and Services Administration (HRSA), have supporting promising primary and secondary HIV/AIDS prevention interventions in African-American communities over the past two decades [18]. As a result, a series of intervention activities were launched to target multiple segments of the population, including African-American women living with HIV/AIDS and HIV-negative women participating in high risk behaviors, including drug use and unprotected sex [10,19,20].

Prevention interventions are commonly carried out by community-based, non-profit organizations and AIDS service organizations targeting African American women. These strategies emphasize the importance of condom use and negotiation, as well as life skills and career development [20,21]. Introducing tailored prevention interventions and building support centers could increase HIV/AIDS knowledge and related capacities towards risk reduction among those at risk or living with HIV/AIDS [20]. Tailored interventions, such as Sisters Informing Sisters about Topics on AIDS (SISTA) and Coping with HIV and AIDS in the Rural Southeast (CHASE), have infused cultural elements towards more effective education and capacity building in safe sex negotiation with partners [22,23,24].

The development of efficacious behavioral interventions is paramount to HIV and STI reduction, but there are relatively fewer interventions targeting high-risk minority population with demonstrable effects. A recent meta-analysis of over 50,000 HIV-related interventions studies published before June 2011 found only 139 (<1%) targeting USA racial minorities [25]. Another review identified only 19% of HIV risk reduction interventions published between 1991 and 2010 targeting African Americans or Hispanics [26]. Existing STI behavioral interventions for African Americans have had only a modest impact in eliminating the HIV burden [24].

Rigorous evaluation of the efficacy of local interventions is critical to the development of replicable and culturally-relevant interventions that reduce HIV/AIDS and hepatitis, particularly among African American women reentering their communities.

### The Intervention

In response to the aforementioned health disparities, Wholistic Stress Control Institute, Incorporated (WSCI) developed and implemented the Health, Enlightenment, Awareness, and Living (HEAL) Intervention for African American formerly incarcerated and homeless women ages 18–55. WSCI, a non-profit community-based organization located in Southwest Atlanta, Georgia, initiated a community-academic partnership with the Morehouse School of Medicine Prevention Research Center (MSM PRC), evaluators of the HEAL intervention [27]. MSM PRC evaluations have been designed to address needs mutually identified by the funder, grantees and target populations to assure that program activities (1) are audience-driven, (2) foster sustained ownership of data collection processes, (3) implement participatory processes, and (4) are perceived as central to program planning, implementation and sustainability. This approach is applied to community-based, regional and national evaluations addressing cancer screening, HIV /AIDS, maternal and child health, mental health, substance abuse, and violence disparities [28,29,30].

The HEAL intervention was designed to address HIV/AIDS and hepatitis risk reduction, incorporating culturally relevant concepts through a life skills enhancement model. Funded by the Substance Abuse and Mental Health Administration (SAMHSA), the overarching goal of the intervention was to build the community’s service capacity to prevent and reduce the onset of substance abuse and transmission of HIV/AIDS and hepatitis among minority homeless and formerly incarcerated populations.

The HEAL Intervention consisted of a 13-session curriculum components addressing substance abuse, hepatitis, and HIV/AIDS, as well as life skills related to parenting, stress management, and vocational assessment towards job readiness. The curriculum was developed based on key components of well-established curricula including: “Living in Balance: Moving from a Life of Addiction to a Life of Recovery” [31], “Beyond Trauma: A Healing Journey for Women” [32], “the HIV/AIDS-SISTA Curriculum” [22,23], and “the Hepatitis Integration Training Manual” (HIT’M) (Table 1) [33,34].

Substance Abuse Prevention: (1) Living in Balance is a research-based intervention that provides a comprehensive, group-oriented framework for people who have substance abuse challenges involving alcohol and other drugs [31]; (2) Beyond Trauma: A Healing Journey for Women promotes a strength-based approach that seeks to empower women and increase their sense of self and uses cognitive-behavioral techniques, expressive arts, and is based on the principles of relational therapy [32].

HIV Prevention: SISTA is a CDC Diffusion of Effective Behavioral Intervention designed to prevent HIV infection among African American women through promoting and reinforcing safe behaviors and strong interpersonal skills in negotiating and sustaining behavior change [22,23].

Hepatitis B and C Prevention: Hepatitis Integration Training Manual (HIT’M) by the American Liver Foundation (ALF) and the New York State Department of Health in collaboration with the CDC Viral Hepatitis Training of Trainers program, is a comprehensive guide for youth and adults that provides Hepatitis B and C education in the areas of HIV/AIDS, sexually transmitted disease, harm reduction and/or drug treatment [33,34].

The HEAL intervention also provided other social and health supports including referrals for substance abuse treatment, transitional housing, adult education, career support, or recovery programs and services. WSCI health educators and community coordinators referred participants for HIV/AIDS and hepatitis B and C screening or hepatitis B immunization to Grady Hospital/Grady Infection Disease Program, the Department of Fulton County Health and Wellness program, AIDS Survival Project, and Mercy Mobile Health Care. WSCI health educators also offered individual assessments for substance abuse and mental health referrals. Participants also received vocational referrals, job placement assistance, and were provided with personal resources upon request (i.e., Bible/prayer partners, hygiene products, dentist referrals). Stress management, coping and relaxation techniques were intentionally and strategically integrated into curriculum to address potential triggers associated with trauma and past experiences. Independent counselors were also available at intervention delivery sites for those needed more extensive supports. Curriculum details are included in Table 1.

## 2. Materials and Methods

### 2.1. Design

A single group repeated measures design was utilized for this study, which consisted of a self-administered HEAL intervention survey comprised of 135 questions assessing participant changes in awareness, knowledge, behavior, and perceptions relative to HIV/AIDS, hepatitis B and C, and substance abuse. At pre-test, participants were assigned a unique code number that allowed for both anonymity in the collection of data and longitudinal participant tracking through to follow-up data collection. Three hundred fifty five (355) pre-test surveys and two hundred twenty eight (228) immediate post-test surveys were administered, representing a 64% response rate. Additionally, participants completed the survey six months after intervention completion; one hundred-ten (110) follow-up surveys were administered, representing a 48% response rate. Those who completed follow-up survey all completed the immediate post-test survey. To reduce attrition, WSCI health educators have kept in contact with participants during and after sessions by calling cell phones, sending emails, providing follow up cards, etc. This was an evaluation study conducted in partnership with those implementing the intervention (WSCI) as detailed earlier. Study was designed for program improvement and was not to be used towards generalizable knowledge but with implications for public health practice as detailed in the sections that follow. Therefore, a determination was made that these activities did not constitute human subject research as outlined by the Office of Human Research Protections guidelines (45CFR 46.102.(d)). Consent forms used to invite participation detailed risks and benefits of the study, as well as contacts for potential triggers that might require further mitigation.

### 2.2. Study Population and Participant Recruitment

Participant eligibility criteria included residence in Fulton and DeKalb counties of Atlanta, GA and self-identification as African American women, age from 18 to 55, who had been previously incarcerated or homeless. The program implementation was conducted from October 2006 to September 2010. The sessions were held twice a week at each site for a total of seven weeks. WSCI health educators utilized a purposive non-probability sampling plan from correctional facilities and community-based sites designed to serve homeless women. WSCI collaborated with correctional facilities, residential treatment facilities, and local community agencies accepting formerly incarcerated and homeless women. Health educators provided the shelters, agencies, and penal facilities with flyers, brochures and information packets that included the interventions curriculum outline and incentives. The site counselor or member of administrative team referred participants to the HEAL intervention continuously on an ongoing basis. If individuals met the eligibility criteria, WSCI health educators offered the opportunity to be enrolled and went through the informed consent process face-to-face to ensure that all questions and concerns associated with participation were addressed. Upon enrollment, participants were grouped in several cohorts. Up to twenty participants, per cohort, met twice a week for 2 hours at residential treatment facilities or at a local agency for formerly incarcerated women. WSCI started a new cohort at least two weeks following intervention completion by a previous cohort. Participants were classified as formerly incarcerated or homeless based on information collected from the host facility by HEAL community coordinators prior to intervention initiation. The intervention had averaged 5 cohorts per year. From 379 participants who were initially invited (114 previously incarcerated and 265 homeless), 355 were enrolled in the study and took the first survey.

Formerly incarcerated populations included adult women being released from prison for project services. The HEAL intervention offered services to formerly incarcerated women at three sites. Two HEAL health educators and one community coordinator worked closely with employees at correctional facilities prior to participants release and with probation officers after participants’ release, to recruit and identify participants for the HEAL intervention. Health educators followed up with formerly incarcerated participants by personal visit and/or phone contact to recruit them once they are in the community. For homeless women, the intervention was advertised in community-based agencies, residential treatment facilities, and shelters designed to serve homeless women. Information cards, brochures, and flyers were distributed to all sites during orientations. WSCI health educators met with participants at community sites to explain the services and to register interested participants. Incentives used to increase intervention retention included a $20 gift card following completion of post-test and a $10 gift card at 6-month follow-up. WSCI and MSM PRC worked collaboratively to obtain written informed consent.

Intervention sessions were delivered by a team of two female African American educators to groups from 10 to 20 participants. Two WSCI health educators had 24 hours of training on HIV/AIDS prevention interventions and relationship development curriculum that comprised of SISTA intervention, SisterLove, Inc., AID Atlanta, and Red Cross. Additionally, WSCI health educators had 24 hours training on stress management utilizing WSCI Stress Management curriculum, and 8 hours training on substance abuse prevention during WSCI in-service trainings and supervision conducted by a Licensed Professional Counselor who has addiction certification. To account for participants’ attendance and intervention session completion, a daily sign-in form was utilized by an internal data manager to record attendance. A maximum of 13 dosages or intervention sessions were offered during the eight-week period. A minimum of eleven sessions were required for program completion determining eligibility for pre-test, immediate post-test and follow surveys. An internal data manager compiled an attendance tracking log to display total attendance for each session for all participants throughout the program duration. This log allowed participants, staff, and evaluators to track missed sessions and overall completion rates for participants.

### 2.3. Measures

The instruments administered were approved and validated by the Government Reporting and Results Act, Performance Assessment Rating Tool, Office of Management and Budget, Substance Abuse and Mental Health Administration (SAMHSA), National Outcome Measures (NOMS), and Minority AIDS Initiative. Intervention outcomes were measured by the NOMS HIV Cohort 6 survey instruments and assessed HIV/AIDS and hepatitis knowledge, perceptions of risky behaviors, and intentions to use condoms [35,36,37,38,39,40].

The outcome domains analyzed for intervention efficacy were:

#### 2.3.1. HIV/AIDS and Hepatitis Knowledge

A series of 18 questions were asked assessing the participants’ knowledge of HIV/AIDS and hepatitis B and C [33,34,35]. Responses included “Yes”, “No”, or “Don’t Know” choices. Correct responses received a score of 1 and incorrect answers a score of 0. These responses were summed to form a two knowledge scores that could range from 0 to 6 for HIV/AIDS and 0 to 12 for hepatitis B and C.

#### 2.3.2. Condom Use

Condom use was measured by series of 6 questions. Participants were asked question about protected vaginal sex “Have you had vaginal sex in the past 30 days?” Responses included “Yes”, “No”, or “Don’t Know” choices. If participant reported sexual activity in last 30 days, the next question was asked: “The last time you had vaginal sex, was it protected or unprotected?” A similar set of questions were asked about oral and anal sex [40].

#### 2.3.3. Sexual Behavior

Participants were asked various questions about their sexual practices in the past 30 days and in the past 3 months. Sexual behaviors included oral, vaginal, anal, or unprotected sex (“Have you had oral sex in the past 30 days? The last time you had oral sex, was it protected or unprotected?”); sex with men, women, or injection drug users; individuals who possibly had an STD or HIV/AIDS (In the past 3 months, have you had unprotected sex (vaginal, anal, or oral) with a partner you know had, or suspected of having a sexually transmitted disease (STD)?); sex in exchange for money, drugs, or shelter (In the past 3 months, have you had unprotected sex (vaginal, anal, or oral) with someone in exchange for money, drugs, or shelter?) or sex under the influence of drugs or alcohol (In the past 3 months, have you had unprotected sex (vaginal, anal, or oral) with someone under the influence of drugs or alcohol?). Participants were also asked about their use of protection the last time they had vaginal, oral, anal sex and about their intentions to use condoms in the next 6 months with responses ranging from 1 (“Not at all likely”) to 4 (“Very Likely”) [40].

#### 2.3.4. Risk Perception

Participants were asked about the risks of unprotected oral, vaginal, and anal sex. The responses were on a scale of 1 (“No Risk”) to 4 (“Great Risk”) [39,40].

#### 2.3.5. Substance Use

Substance abuse was measured by a series of 10 questions. Participants were asked about the number of days in the last 30 days that they used substances, including cigarettes, tobacco, alcohol, marijuana, illegal drugs, crack/cocaine, prescription drugs, injected drugs, and methamphetamines [40].

### 2.4. Statistical Analysis

Matched pre-test, post-test, and follow-up surveys were utilized for analysis.A few participants did not complete all questions on the 135 item survey; hence, missing data were excluded from the analysis. Pearson’s chi-square test, paired-sample t-test, McNemar, and ANOVA tests were used to assess change among intervention objectives. Data analysis was conducted using PASW Statistics for Windows, Version 18.0 (SPSS, Inc., 2009, Chicago, IL, USA).

We performed a posthoc retrospective power calculation for sample of 228 participants matched of pre-post and immediate post-test, mean difference 0.5, within group standard deviation 3, and alpha levels 0.05 for paired T test. Our calculation resulted in 70.8% power of this study. Additionally, we calculated a post-hoc retrospective power calculation for matched pre-test, post-test and follow up sample of 110 participants, mean difference 0.8, within group standard deviation 3 and alpha levels 0.05 for paired T test. Data indicate that the difference in the response of matched pairs is normally distributed with standard deviation 3. Our calculation resulted in 79.2% power. We used PS software to calculate power of the study [41].

## 3. Results

### 3.1. Participant Characteristics

Our analytic sample comprised 355 pre-test surveys, 228 immediate post-test surveys, and 110 follow-up surveys. On pre-test, 105 adults were formerly incarcerated and 250 homeless. On pre-test (n = 355), participants ranged in age from 19 to 55, with a mean age of 37.7. Most (80.7%) participants described their ethnicity as African-American, a little over half of participants reported completing grade 12 or higher (62.6%), and most (81.1%) reported a household income of $10,000 or less. About one-third (34.8%) reported being homeless at the time of survey administration. Table 2 provides details on the characteristics of participants by formerly incarcerated and homeless status.

### 3.2. HIV/AIDS and Hepatitis B and C Knowledge

Prior to the intervention, among paired pre-test and post-test surveys, the mean HIV/AIDS score was 4.79 and after the intervention the mean score was 4.94. Though this resulted in a 3.1% increase in knowledge it was not statistically significant. This may be explained by a relatively high level of baseline knowledge. The mean number of correct responses to the twelve Hepatitis items at pre-test was 5.97 and increased to 7.04 at immediate post-test survey. This increase of 17.9% was also statistically significant (t (116) = −4.62, *p* < 0.001).

When examining differences in knowledge over time, there was a significant change in hepatitis B and C knowledge from pre-test, to immediate post-test, and follow-up knowledge scores (*F* (1.88) = 17.631, *p* < 0.001).Post hoc tests indicate that hepatitis B and C scores at post-test were significantly greater than pre-test scores (*p* < 0.001) and six-month follow-up scores were significantly greater than pre-test scores (*p* < 0.001). HIV/AIDS scores also significantly differed over time (*F* (1.94) = 3.177, *p* < 0.05) using Greenhouse-Geisser (chi square = 2.37, *p* > 0.05). However, post hoc tests revealed that only differences from pre-test to post-test were statistically significant (*p* < 0.05) (Table 3).

### 3.3. Condom Use

Participants were asked about their use of protection the last time they had vaginal, oral, or anal sex. Of those who reported having vaginal sex in the past 30 days, reported condom use increased by 12.0% from pre-test to post-test survey, however the increase was not significant (McNemar test *p* = 0.478). Among those who reported having oral sex in the past 30 days, condom use increased by 2.6% from pre-test to immediate post-test survey (McNemar test *p* = 0.655). Raw numbers reflecting self-reported anal sex were extremely low and account for the large percentage decrease. Condom use continued to show an increase over time with protected anal (35.9%), vaginal (17.2%) (McNemar test *p* = 0.655), and oral (13.9%) (McNemar test *p* = 0.317) sex demonstrating increases from pre-test to follow-up survey (Table 4).

### 3.4. Sexual Behavior

All forms of sexual behaviors decreased from pre-test to immediate post-test survey. Significant decreases were indicated for the number of participants who: had unprotected sex in exchange for money, drugs or shelter (6.1%) (McNemar test *p* = 0.008), had unprotected sex with a partner having an STD (3.6%) (McNemar test *p* = 0.039), and had sex while under the influence of drugs or alcohol (14%) (McNemar test *p* < 0.001) from pre-test to immediate post-test survey.

When examining risky sexual behaviors over time, HEAL Intervention participants demonstrated a decrease in unprotected sex with partners having an STD (McNemar test n = 196, *p* = 0.039). For unprotected sex in exchange for money, drugs or shelter and sex under the influence of drugs or alcohol, decreases were statistically significant from pre-test to immediate post-test (McNemar test n = 196, *p* = 0.008 and n = 196, *p* < 0.001) and from pre-test to follow-up survey (McNemar test n = 76, *p* = 0.039 and n = 73, *p* = 0.007) (Table 4).

### 3.5. Sexual Risk Perceptions

At pre-test, all forms of unprotected sex were seen as “Moderately Risky”, with pre-test means falling between 3 (“Moderate Risk”) and 4 (“Great Risk”) with 3.72 for vaginal sex, 3.57 for oral sex, and 3.81 for anal sex. Most perceptions shifted towards the favorable direction of 4 or “Great Risk” including: unprotected vaginal sex (immediate post-test *M* = 3.75) (t (195) = −0.52, *p* = 0.603); unprotected oral sex (immediate post-test *M* =3.63) (t (193) = 0.91, *p* = 0.362); unprotected anal sex (immediate post-test *M* = 3.85) (t (195) = −1.04, *p* = 0.300); and sex with drug use (immediate post-test *M* = 3.68) (t (196) = −1.11, *p* = 0.270). However, sex with alcohol use (pre-test *M* =3.60, immediate post-test *M* =3.59) showed a slight mean decrease (t (196) = 0.09, *p* = 0.932) (Table 3).

### 3.6. Substance Use

The results showed a significant decrease in the mean number of days of cigarette (t (183) = 4.63, *p* < 0.001),tobacco (t (184) = 3.10, *p* = 0.002), alcohol (t (195) = 2.55, *p* = 0.011), being very drunk (t (193) =2.74, *p* = 0.007), marijuana (t (201) = 2.57, *p* = 0.011), illegal drug (t (198) = 3.16, *p* = 0.002), and cocaine/crack (t (197) = 2.98, *p* = 0.003) use from pre-test to immediate post-test survey. Overall, the mean number of days of self-reported methamphetamine, prescription drug, and injected drug use in the past 30 days also decreased during this period (Table 3).

Mean number of days of substance use was also examined over time, comparing pre-test, immediate post-test, and six-month follow-up data for adult participants. The results show that the mean number of days for the use of tobacco products (*F* (1.847) = 6.70, *p* = 0.002) among pre-test, immediate post-test, and follow-up time points differed significantly. Post hoc tests revealed that for tobacco products, the pre-test mean was significantly higher than the immediate post-test mean (*p* = 0.022) as well as the follow-up mean (*p* = 0.010). Overall, comparing aggregate pre-test and follow-up means, mean number of days of substance use continued to show a decrease from pre-test to follow-up for all substances.

In addition to the outcomes demonstrated by survey results, HIV testing behavior was also monitored for HEAL Intervention participants. Over the span of intervention implementation, 138 women were tested for HIV. Of this number, two women tested positive for HIV and were subsequently referred for medical treatment. Furthermore, among 212 women that were screened for hepatitis B or C, 5 women admitted to have positive results for hepatitis C and were subsequently referred for medical treatment.

## 4. Discussion

The HEAL intervention demonstrated the efficacy of the intervention for HIV/AIDS prevention and decrease associated risk factors, including substance abuse and unprotected sex in African American women, ages 18–55, residing in Atlanta, GA. Participants demonstrated statistically significant increases in the percentage of hepatitis B and C knowledge over time. The percentage of protected oral and vaginal sex had increased after the intervention though changes did not reach statistical significance. Results also indicated statistically significant decreases in unprotected sex in exchange for money, drugs or shelter and sex under the influence of drugs or alcohol. Substance use decreased for all categories, with statistically significant decreases in the mean number in the past 30 days for the use of cigarettes, tobacco products, alcohol, marijuana, illegal drugs, and crack/cocaine.

The HEAL Intervention also facilitated HIV and hepatitis screening and referral for treatment in community outreach settings, representing a multilevel approach that targeted both the individual participants and the context within which critical supports were needed. Recent research shows that HIV testing is important in identifying undiagnosed infections and is a critical complement to interventions that increase knowledge, awareness and capacity building toward individual risk reduction [42]. Delays in diagnosis result in lost opportunities for prevention and treatment, resulting in poorer health outcomes, further exacerbating racial and ethnic HIV/AIDS disparities [43].

Beyond the positive implications of results described above, additional strengths of this study are noteworthy. First, a satisfactory response rate (48%) to a 135-item survey with very sensitive and personal questions was observed. This is likely attributable to WSCI health educators who were socially and contextually congruent to participants. More specifically, health educators had a combined 45 years of planning and implementation in gender specific programming for incarcerated, homeless and high-risk female populations. Their experience represented prior work in the capacity of a health educator or counselor for numerous social service programs designed for indigent populations including the homeless and formerly incarcerated female adults and those in substance abuse recovery. They also offered on-going case management and relationship building to assuage participants’ concerns regarding disclosure of responses. Second, confidential survey administration allowed for pre-test and post-test matching by non-personal identifiers with results that were likely stronger than if anonymous survey administration had been implemented. The intervention’s 6-month follow-up supported longer-term assessment of intervention effectiveness [44]. Finally, the addition of HIV/AIDS and hepatitis screening and referral represented a multilevel approach, including both individual and systems linkages toward secondary and tertiary HIV/AIDS prevention and risk reduction.

Limitations of this intervention are also acknowledged. All surveys administered represented participants’ self-report which is subject to several bias including: selective memory, telescoping (i.e., recalling events that occurred at one time as if they occurred at another time), attribution (i.e., the act of attributing positive events and outcomes to one’s own agency while attributing negative events and outcomes to external forces), and exaggeration [45]. Another limitation of the intervention was a post-hoc power calculation. While ideally power should be conducted prospectively, our retrospective calculation indicated that the study had a sufficient power. In addition, while the strength of 6-month follow-up is aforementioned, lower response rates were observed. Differences in the intervention sites and populations served may also contribute to the findings. This issue could have been addressed through sub-group analysis, however due to insufficient sample size sub-group analysis was not conducted. Finally, the long survey instrument (135 items) may have served as a potential barrier to completion.

## 5. Conclusions

The HEAL Intervention reflects an evidence-based community intervention that was culturally designed for African American women. Findings point to strengths of integrating HIV/AIDS, substance abuse, and hepatitis knowledge with discussion of consequential outcomes associated with risk taking behaviors. These elements were complemented by other components including life skills development, stress management, group and facilitator relationship building and an open environment. Results hold implications for the design of community-based interventions and the community-campus partnerships that assess them.

## Figures and Tables

**Table 1:** Health, enlightenment, awareness, living (HEAL) intervention curriculum key components.

Session Topics	Curriculum	Learning Objectives
1. Introduction	Pre-test Survey AdministrationOrientation /Stress Management	1. Learn to identify the causes, signs and ways to manage stress. 2. Practice relaxing techniques.
2. HIV	Positive affirmation to overcome feelings of anxiety and fear Relaxation Exercise Sista:*Ethnic/Gender Pride; HIV/AIDS Education*	1. Practice relaxing techniques. 2. Recognize the pride that exists within Black women. 3. Identify and discuss sources of pride for Black women.
3. Substance Abuse	Positive affirmation to overcome feelings of loneliness and sadness Relaxation Exercise *Living in Balance:* Substance abuse and Trauma; Effect ofDrugs and Alcohol on Sex *Beyond Trauma:* The Connection between Violence, Substance Abuse and Trauma; The Addiction and Trauma Connection (addiction and recovery & trauma and healing)	1. Practice relaxing techniques. 2. Define and identify the impact of substance abuse and addiction. 3. Learn to identify abuse and trauma in women’s lives. 4. Learn coping skills.
4. Hepatitis	Positive affirmation to overcome feelings of anxiety and fear Relaxation Exercise *Hit’em:* Hepatitis Presentation	1. Practice relaxing techniques. 2. Learn definition and terms related to hepatitis. 3. Described routes of hepatitis B and C transmission, signs of infection, prevention, and co-infections.
5. HIV	Positive affirmation to overcome feelings of anger Relaxation Exercise *Sista:* Assertiveness Skill Training; Behavioral Self-Management promoting decisions; Coping Skills	1. Practice relaxing techniques. 2. Enable participants to learn differences between being assertive, aggressive, and non-assertive. 3. Learn to identify the six steps in good decision making. 4. Increase the women’s ability to use healthy decisions. 5. Understand coping and how to deal with negative feedback.
6. Substance Abuse	Positive affirmation to overcome feelings of loneliness and sadness Relaxation Exercise *Living in Balance:* Alcohol and Other Drug Education (stimulants, hallucinogens, antidepressants and their effects on mental function) *Living in Balance:* Triggers of Substance Abuse (definition and examples of triggers, effect and causality of relapse, & prevention)	1. Practice relaxing techniques. 2. Learn to identify the effects of substance abuse on lives. 3. Learn to identify triggers, relapses, and how to stop leading to relapse.
7. Hepatitis	Positive affirmation to overcome feelings of anxiety and fear Relaxation Exercise *Hit’em:* Hepatitis Presentation (Observation conducted by Evaluators on Hepatitis)	1. Practice relaxing techniques. 2. Learn definition and terms related to hepatitis. 3. Describe routes of hepatitis B and C transmission, signs of infection, prevention, and co-infections.
8. HIV	Positive affirmation to overcome feelings of loneliness and sadness *Sista:* Review of the topics: Ethnic/Gender Pride; HIV/AIDS Education; Assertiveness Skill Training; Behavioral Self-Management promoting decisions; Coping Skills	1. Practice relaxing techniques 2. Review Session materials covered in *SISTA Sessions 1–5.*
9. Substance Abuse	Positive affirmation to overcome feelings of anger Relaxation Exercise *Living in Balance:* Anger and Communication (understanding anger and improving communication) *Beyond Trauma:* Mind and Body Connection(emotional wellness); The World of Feelings (common feelings)	1. Practice relaxing techniques. 2. Learn to understand anger and how to express anger. 3. Learn to understand emotional wellness. 4. Learn to express and communicate feelings. 5. Learn to recognize and share feelings. 6. Learn empathy and compassion.
10. Substance Abuse	Positive affirmation to overcome feelings of anger Relaxation Exercise *Living in Balance:* Spirituality (what it is spirituality, relationship to spirituality and drug abuse, different paths and tips to increase spirituality and prayer) *Beyond Trauma:* Spirituality (learning to connect on different levels and learning to care for self); Meditation; Self Soothing (Relaxation); Healthy Relationships (Observation conducted by Evaluators on Substance Abuse)	1. Practice relaxing techniques. 2. Defining higher power and using prayer. 3. Learn about Self Soothing and healthy relationships.
11. Review	Positive affirmation to overcome feelings of anxiety and fear Relaxation Exercise Review	1. Review stress management techniques learned. 2. Review Session materials covered in *Siesta, Living In Balance, Beyond Trauma, and Hit’em.*
12. Review	Positive affirmation to overcome feelings of anxiety and fear Relaxation Exercise Review	1. Review stress management techniques learned. 2. Review Session materials covered in *SISTA, Living In Balance,0Beyond Trauma, and Hit’em.*
13. Testing and Graduation	Relaxation Exercise Posttest Survey Administration Focus Group Conducted by Evaluator Graduation Ceremony	1. Practice Stress Management Exercise.

**Table 2:** HEAL intervention participant demographic characteristics.

Characteristic	Overall		Homeless		Formerly Incarcerated	
	n	%	n	%	n	%
Total	355	100%	250	70.4	105	29.6
Age (Mean, SD)	36.8 (9.96)		35.9 (9.94)		38.9 (9.72)	
Black/African American	272	77.3%	190	76.9%	82	78.1%
White	56	15.6%	36	14.6%	19	18.1%
American Indian	3	0.9%	2	0.8%	1	1.0%
Hawaii	3	0.9%	3	1.2%	0	0.0%
Asian	3	0.9%	3	1.2%	0	0.0%
Hispanic	4	1.1%	4	1.6%	0	0.0%
Other	12	3.4%	9	3.6%	3	2.7%
Any Children	313	89.4%	220	89.4%	93	89.4%
Single	195	55.4%	139	56.0%	56	53.8%
Completed 12th grade or higher	221	62.6%	160	64.3%	61	58.7%
Annual Income < $10,000	275	81.1%	198	81.1%	77	81.1%
Unemployed	309	89.3%	229	93.5%	80	79.2%
Homeless	122	34.4%				

**Table 3:** HEAL program participant outcomes at pre-test, immediate post-test, and follow-up survey.

	Pretest		Posttest ^a^		Pretest Posttest	Follow-up ^b^		Posttest Follow-up
	Mean SD		Mean SD	n	*p* Value	MeanSD	n	*p* Value
**Knowledge**								
HIV Knowledge	4.79 (1.08)		4.94 (0.99)	225	0.168	4.75 (1.16)	109	0.680
Hepatitis Knowledge	5.97 (2.68)		7.04 (2.73)	220	<0.001	7.45 (2.99)	106	<0.001
**Sexual Risk Perception**								
Risk of unprotected vaginal sex	3.72 (0.76)		3.75 (0.72)	197	0.603	3.70 (0.79)	85	0.342
Risk of unprotected oral sex	3.57 (0.84)		3.63 (0.81)	195	0.362	3.55(0.91)	86	0.228
Risk of unprotected anal sex	3.81 (0.59)		3.85 (0.54)	197	0.300	3.81(0.66)	84	0.297
Risk of sex while under the influence of drugs	3.62 (0.78)		3.68 (0.72)	198	0.270	3.73 (0.74)	85	0.170
Risk of sex while under the influence of alcohol	3.60 (0.78)		3.59 (0.77)	198	0.932	3.68 (0.76)	83	0.389
**Substance Use**								
Smoke a cigarette	8.04 (12.6)		5.01(10.81)	183	<0.000	4.48(10.1)	75	0.067
Use other tobacco products	2.84 (8.06)		1.05 (5.04)	184	0.002	0.66 (3.86)	79	0.002
Drink one or more drinks of an alcoholic beverage	1.26 (4.44)		0.39(1.63)	195	0.011	0.58(2.60)	83	0.2100
Been drunk or very high from drinking alcoholic beverages	0.61 (2.94)		0.03 (0.19)	193	0.007	0.14 (0.68)	81	0.4346
Use marijuana or hashish	1.27 (5.40)		0.42 (3.10)	201	0.011	0.43 (2.78)	86	0.670
Use any other illegal drug	1.24 (5.35)		0.05 (0.42)	198	0.002	0.03 (0.23)	85	0.035
Use cocaine or crack	0.94 (4.43)		0.00 (0.00)	197	0.003	0.00 (0.00)	84	0.068
Use methamphetamine	0.18 (1.36)		0.15 (2.13)	198	0.867	0.00 (0.00)	86	0.125
Used prescription drugs to feel good or get high	0.37 (3.04)		0.09 (1.28)	199	0.232	0.07 (0.58)	86	0.184
Injected any drugs	0.04 (0.36)		0.00 (0.00)	202	0.117	0.00 (0.00)	87	0.321

**^a^** Results for immediate posttest survey reflect those from matched pre-test post-test surveys; **^b^** Results for follow-up survey reflect those from matched pre-test post-test follow-up surveys.

**Table 4:** HEAL program participant outcomes at pre-test, immediate post-test, and follow-up survey.

	Pretest		Posttest ^a^		Pretest Posttest	Follow-up ^b^		Posttest Follow-up
	n	%	n	%	*p* Value	n	%	*p* Value
**Condom Use ^c^**								
Protected Vaginal Sex	40	36%	30	48.4%	0.478	15	53.6%	0.655
Protected Oral Sex	14	17%	9	20.5%	0.655	7	31.8%	0.317
Protected Anal Sex	4	30%	1	25.0%	n/a	4	66.7%	n/a
**Sexual Behavior**								
Unprotected sex in exchange for money, drugs, or shelter	20	10%	8	4.0%	0.008	1	1.3%	0.039
Unprotected sex with a partner having a STD	9	4.4%	2	1.0%	0.039	1	1.3%	0.375
Unprotected sex with a partner having HIV/AIDS	2	1.0%	2	1.0%	1.000	1	1.3%	0.564
Unprotected sex with an injected drug user past	7	3.4%	2	1.0%	0.180	5	6.6%	0.706
Had sex while under the influence of drugs or alcohol	48	24.1%	20	10.0%	<0.001	3	4.0%	0.007

**^a^** Results for immediate posttest survey reflect those from matched pre-test post-test surveys; **^b^** Results for follow-up survey reflect those from matched pre-test post-test follow-up surveys; **^c^** Condom Use: Out of individuals who reported sexual activity within the past 30 days.
